# Hyperthermia in Term Neonates: Profile and Factors Associated With Sepsis

**DOI:** 10.7759/cureus.107435

**Published:** 2026-04-21

**Authors:** Shivangi R Jaiswal, Ruchi Rai, Dharmendra K Singh, Shivi Singh

**Affiliations:** 1 Department of Pediatrics, Postgraduate Institute of Child Health, Noida, IND; 2 Department of Neonatology, Postgraduate Institute of Child Health, Noida, IND; 3 Department of Pediatrics, Dr. Vaishampayan Memorial (VM) Government Medical College, Solapur, IND

**Keywords:** dehydration fever, environmental hyperthermia, febrile neonates, infant lethargy, sepsis

## Abstract

Aim of the study

The aim of this study was to distinguish between hyperthermia (HT) due to sepsis and environmental hyperthermia (EH) or dehydration fever (DF). Hyperthermia (HT) in neonates may be due to many reasons, common among them being environmental hyperthermia (EH), dehydration fever (DF), and sepsis. It is important to make a provisional diagnosis at presentation for better outcomes and prevent the indiscriminate use of antimicrobials. We therefore decided to study the profile of term neonates with HT and analyze which clinical or laboratory parameters are associated with the diagnosis of sepsis.

Methods

We enrolled term neonates admitted with HT (axillary temperature: > 37°C/99.5°F). Complete blood count, C-reactive protein (CRP), blood culture, and other relevant investigations were done for enrolled newborns. Well-appearing babies were kept under close observation and supportive care. On the occurrence of a repeat episode of HT, antibiotics were started, and a lumbar puncture was done. Babies who were not well-appearing were started on antibiotics at admission. Antibiotics were stopped in neonates who were well at 48-72 hours and had a sterile blood culture. All neonates were divided into three groups: EH, DF, and sepsis. The characteristics of neonates in the three groups were compared, and attributes associated with the diagnosis of sepsis were analyzed.

Results

We enrolled 90 term neonates over a period of 18 months. Sepsis, EH, and DF accounted for 42.2%, 34.5%, and 23.3% of the cases, respectively. Mean C-reactive protein (CRP) levels were significantly higher in the sepsis group. The presence of lethargy at admission, a repeat episode of HT, and elevated CRP were significantly associated with the diagnosis of sepsis.

Conclusion

All neonates with HT do not need to be treated as sepsis. Babies who are sick at presentation must be promptly treated with antibiotics. Well-appearing neonates with HT may be kept under close observation. A repeat episode of HT in a well-appearing neonate warrants starting antibiotics and thorough evaluation. A scoring system for the evaluation of neonates needs to be developed and validated.

## Introduction

Temperature maintenance has always been of foremost concern and extremely crucial for the management of newborns. Both hypothermia and hyperthermia (HT) pose a challenge for the management of a newborn. They increase the morbidity and mortality in newborns [1,2]. The World Health Organization (WHO) identifies that HT can occur just as easily as hypothermia and can be equally dangerous [3].

HT in neonates is defined as axillary temperature of >37°C/99.5°F by the World Health Organization (WHO). HT leads to increased basal metabolic rate, water loss, and ultimately dehydration. It causes flushing, hypotonia, abnormal posture, vasodilatation, and hypotension. It can cause irritability and then lethargy, even shock, convulsions, and coma. The thermal stress can increase the risk of sudden infant death syndrome (SIDS) directly via lethal HT or indirectly by altering autonomic functions [4].

HT occurs when the body temperature increases without a change in temperature set point in the hypothalamus; in contrast, fever occurs when the body’s temperature set point is higher [5]. In fever due to sepsis, the extremities may remain cold despite the axillary temperature being high. In environmental hyperthermia (EH), the extremities are also warm or hot [6]. However, clinically, it is difficult to differentiate between fever and HT. We will be using the term HT for both entities.

HT may be caused by relatively high environmental temperature, infection, dehydration, CNS dysfunction, or medication [7]. A study by Janakiraman et al. on the clinical profile of febrile neonates found culture-positive sepsis to be present in 16% of newborns, although 63% had a positive sepsis screen [8]. Environmental hyperthermia (EH) and dehydration fever (DF) constituted 1% and 36% of the total cases, respectively. EH is common in neonates as they do not sweat and have immature temperature regulation mechanisms; therefore, they are at a higher risk of developing HT when faced with high ambient temperature [9].

Sepsis may sometimes present with fever, although it is not a common symptom of sepsis in neonates. Sepsis is difficult to rule out and associated with high morbidity and mortality; therefore, most clinicians would administer antibiotics in all cases of HT in neonates. This leads to the indiscriminate use of antimicrobials and may not be appropriate.

Dehydration fever (DF) is another known cause of HT in neonates. This is seen in healthy term neonates in the first week of life, usually on exclusive breastfeeding [10]. HT in low-risk term infants and with no other symptoms during the initial few days is most likely to be related primarily to dehydration, breastfeeding, and Caesarean delivery and less likely to be due to infection [11].

Identifying febrile neonates with sepsis is a diagnostic challenge. The prompt identification and treatment of sepsis are crucial in neonates. It is equally essential to identify neonates who are less likely to have sepsis. There is a paucity of guidelines regarding the management of such neonates in lower-middle-income countries (LMICs). There is always a dilemma whether to start antibiotics in such newborns. Most studies have focused on the etiology and management of febrile young infants of less than three months of age. Therefore, we planned to study term neonates presenting with HT to evaluate the different causes. We aimed to analyze the clinical features and laboratory parameters associated with different causes of HT in a neonate.

## Materials and methods

This study was a prospective cohort study conducted in the neonatal intensive care unit (NICU) of a tertiary-level teaching institute between 1 July 2022 to 31 December 2023 over a period of 18 months. The study was approved by the Institutional Ethics Committee of Super Speciality Paediatric Hospital and Postgraduate Teaching Institute (approval number: 2022-06-IM-26). All newborns fulfilling the inclusion criteria were enrolled in the study after taking consent from one of the parents. The inclusion criteria were as follows: babies born at term and admitted to the NICU with an axillary temperature of >37.5°C or 99.5°F at the time of admission. The exclusion criteria were as follows: (i) newborns with a history of hospital stay within the last seven days, (ii) a history of antibiotic administration within seven days of admission, or (iii) any obvious signs of infection, such as abscess, cellulitis, septic arthritis, mastitis, and otitis media.

We enrolled all term neonates with documented HT at admission. HT was defined as axillary temperature of >37.5°C or 99.5°F. The axillary temperature was measured by a digital thermometer in the dry axilla of the newborn. The baby was thoroughly assessed, and a detailed history was taken. History included the demographic and birth history, the type of feeding, previous hospitalization, and other complaints. The baby was thoroughly examined, and immediate care was given as per the baby’s requirements.

All enrolled neonates underwent a workup, which included complete blood count, blood culture, C-reactive protein (CRP), blood sugar, kidney function tests, and serum electrolytes. Other investigations were done as needed. Blood culture was taken by venipuncture after proper cleaning and disinfecting the site under aseptic conditions. A sample of 1 mL of blood was withdrawn and sent in an automated blood culture bottle to the microbiology laboratory immediately.

**Figure 1:**
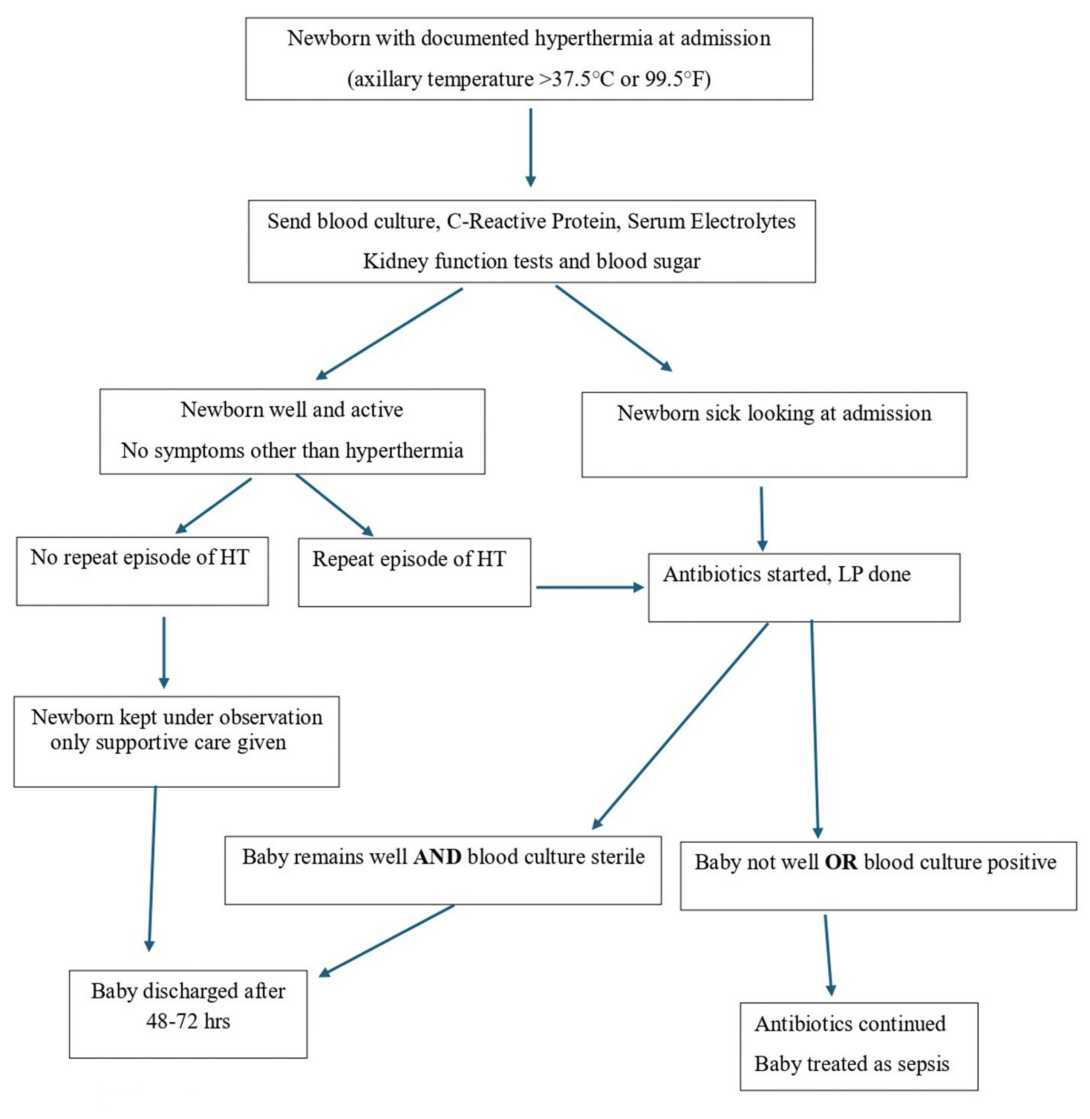
Algorithm for the approach and management of neonates with hyperthermia (HT) LP: lumbar puncture

A well-appearing baby who was active and feeding well was given supportive care, including temperature maintenance, and was kept under close observation. If such a baby developed a second episode of HT in the NICU, empirical antibiotics were started (injection ampicillin and injection gentamicin), and a lumbar puncture was done (Figure 1). A lumbar puncture was done under aseptic precautions, and the cerebrospinal fluid (CSF) was sent for total and differential cell count, protein, sugar, and culture. The further course of management was decided on the laboratory reports and clinical condition.

A baby who had any signs of sickness at admission was started on antibiotics, and cerebrospinal fluid (CSF) examination was done. The blood culture was withdrawn before the first dose of antibiotics was administered. The baby was managed according to the laboratory reports and their clinical condition. Antibiotics were stopped in neonates if the culture reports were sterile and the baby was well at 48-72 hours. Babies were treated with appropriate antibiotics for 5-7 days for clinical sepsis, 14 days for culture-positive sepsis, and 21 days for meningitis. Babies who were not started on antibiotics or in whom antibiotics were stopped for no evidence of sepsis were observed for at least 48 hours after stopping antibiotics before discharge.

Babies were stratified into three groups based on the final diagnosis: EH, DF, or sepsis. The following definitions were used for the final diagnosis.

Dehydration fever

A newborn was diagnosed as having dehydration fever if it had all the following: (i) age of <8 days, (ii) exclusively/predominantly breastfed (75%), (iii) sterile blood culture, (iv) weight loss of ≥10% from birth weight, and (v) features of dehydration.

Environmental hyperthermia

A newborn was diagnosed with environmental HT if all the following were fulfilled: (i) showed rapid improvement after admission, (ii) had a sterile blood culture, and (iii) did not have features suggestive of DF.

Sepsis

Babies with clinical sepsis, culture-positive sepsis, and meningitis were labelled as sepsis. Clinical sepsis occurs when the blood culture is sterile but clinical features are suggestive of sepsis. Meningitis was defined as the presence of any of the following: CSF protein of >170 mg/dL or cells of ≥ 30 cells/mm^3^ or a growth in the CSF. Culture-positive sepsis occurs when the blood culture shows growth of a pathogenic bacteria/fungus. The winter months were taken from October to February, and the summer months were March to September. CRP of >10 mg/mL was considered elevated.

Statistical analysis

The statistical analysis was done using the Epi Info 7.2.6.0 software (CDC, Atlanta, GA). The numerical values were expressed as mean (standard deviation {SD}) for normally distributed data and median and interquartile range (IQR) for non-normally distributed data. Qualitative data were represented as proportions and percentages. ANOVA and the Kruskal-Wallis test were applied to compare numerical data between the three groups (EH, DF, and sepsis). A p-value of less than 0.05 was considered significant. Multivariate logistic regression analysis was used to find the association between characteristics and the risk of sepsis.

## Results

We enrolled 90 babies admitted to the NICU with HT during the study period. The mean (SD) birth weight of the newborns was 2562 g (±522 g), most were boys (57.7%) and delivered vaginally (86.7%). The mean (SD) temperature at admission was 38.0°C (±0.7°C); the maximum temperature recorded was 40.7°C. Table 1 shows the numbers and percentages of the attributes found in the enrolled newborns. Sepsis was seen in 38 (42.2%) neonates, EH in 31 (34.5%), and DF in 21 (23.3%).

**Table 1:** Characteristics of neonates admitted with hyperthermia Data are presented as the number of cases (N) with percentage (%) for categorical variables and mean ± standard deviation (SD) and median (interquartile range {IQR}) for continuous variables. Percentages are calculated based on the total study population (N = 90) M, male; F, female

Characteristics	N (%)/mean ± SD/median (IQR)
M/F	1.3:1
Vaginal delivery, n (%)	78 (86.7)
Age at admission (days), median (IQR)	5 (2,14)
Birth weight (g), mean (SD)	2562 ± 522
Hospital stay (days), median (IQR)	4.5 (2,14)
Exclusive breastfeeding, n (%)	67 (74.4)
Low birth weight, n (%)	43 (47.8)
Temperature at admission (°C), mean (SD)	38.3 ± 0.7
Severe hyperthermia (>39°C), n (%)	19 (21.1)
Cases in summer months, n (%)	57 (89) (N = 64)
Diagnosis	
Environmental hyperthermia, n (%)	31 (34.5)
Dehydration fever, n (%)	21 (23.3)
Sepsis, n (%)	38 (42.2)
Culture-positive sepsis, n (%)	07
Clinical sepsis, n (%)	17
Meningitis, n (%)	19
Outcome	
Discharge, n (%)	87 (96.6)
Death, n (%)	3 (3.4)

We discharged 87 babies (96.6%), and three (3.4%) babies died. All three babies who expired were top-fed and were diagnosed with sepsis. One of these babies had *Acinetobacter* septicemia and associated meningitis. Antibiotics were started in 62 babies for the suspicion of sepsis, out of which we stopped antibiotics in 24 neonates whose culture was sterile and who were well at 48-72 hours. In the rest of the 38 babies, no antibiotics were given, and they were kept under observation.

As the duration of the study was 18 months, the data for one year from April 2022 to March 2023 were considered when the analysis of cases, as per the summer and winter months, was done. The summer months accounted for 89% (N = 57) of cases. There was only one case of DF and no case of EH in the winter months.

None of the babies included in the study were readmitted after discharge. Blood culture was sterile in all babies, with only a single episode of HT documented at admission. Culture-positive sepsis was seen in seven babies (*E. coli*,* *two; *Enterobacter cloacae*, two; *Klebsiella pneumoniae*, one; *Acinetobacter baumannii*, one; and *Candida albicans*, one). Meningitis was diagnosed in 19 babies based on cell count and protein levels; CSF was sterile in all these neonates diagnosed with meningitis.

The presence of lethargy, a repeat spike of HT after admission, and elevated CRP were significantly associated with the diagnosis of sepsis. Age at admission was significantly different in the EH, DF, and sepsis groups. Mean serum sodium levels were significantly higher in the DF group, and mean CRP levels were significantly higher in the sepsis group. Table 2 and Table 3 show a comparison of characteristics in newborns with sepsis, EH, and DF.

**Table 2:** Comparison of categorical characteristics among hyperthermic neonates with EH, DF, and sepsis Data are presented as the number of cases (N) with percentage (%) for all categorical variables. The chi-square (χ^2^) test and Fisher’s exact test were used for categorical comparisons. A p-value of <0.05 was considered to be significant EH, environmental hyperthermia; DF, dehydration fever; AKI, acute kidney injury; CRP, C-reactive protein; HT, hyperthermia

Characteristics, N (%)	EH (N = 31)	DF (N = 21)	Sepsis (N = 38)	Test statistic	P-value
Male gender	15 (48.4)	10 (47.6)	13 (34.2)	χ^2^ = 1.73	0.42
Vaginal delivery	28 (90.3)	19 (90.5)	31 (81.6)	χ^2^ = 1.47	0.47
Low birth weight	14 (45.1)	11 (52.3)	18 (47.3)	χ^2^ = 0.26	0.87
Severe hyperthermia	6 (19.3)	4 (19.0)	9 (23.6)	χ^2^ = 0.26	0.87
AKI	6 (19.3)	9 (42.8)	11 (28.9)	χ^2^ = 3.36	0.18
Elevated CRP	11 (35.5)	5 (23.8)	24 (63.1)	χ^2^ = 10.01	0.006
Lethargy	1 (3.2)	1 (4.7)	12 (31.7)	χ^2^ = 8.26	<0.001
Repeat episode of HT	7 (22.5)	2 (9.5)	24 (63.1)	χ^2^ = 20.58	<0.001

**Table 3:** Comparison of numerical variables among hyperthermic neonates with EH, DF and sepsis Data are presented as mean ± standard deviation (SD) for normally distributed continuous variables and median (interquartile range {IQR}) for non-normally distributed continuous variables. The ANOVA test/Kruskal-Wallis test was used for numerical variables. A p-value of <0.05 was considered to be significant *Median (IQR) ANC, absolute neutrophil count; CRP, C-reactive protein; DF, dehydration fever; EH, environmental hyperthermia

Characteristics, mean (SD)	EH	DF	Sepsis	F statistic	P-value
Age at admission (days)*	11 (2,16)	3 (2,5)	7.5 (3,16)	8.02	0.01
Birth weight (g)	2478 ± 492	2607 (455)	2588 (586)	0.32	0.76
ANC (/mm^3^)	5389 ± 2415	3897 ± 2373	5647 ± 4576	1.04	0.35
Serum creatinine (mg/dL)	0.87 ± 0.4	0.91 ± 0.4	1 ± 0.8	1.08	0.34
Serum urea (mg/dL)*	38 (19,47)	62 (44,87)	37 (15,80)	7.73	0.02
Serum sodium (mEq/L)	135.8 ± 5.8	146.1 ± 5.6	139.9 ± 9.1	12.43	<0.001
CRP (mg/dL)*	4.5 (1.5,16.7)	4.1 (1.9,15)	20.8 (3.9,42.7)	9.8	0.007

Table 4 shows the multivariate logistic regression analysis of factors associated with sepsis. The presence of lethargy, the repeat spike of HT, and elevated CRP had a relative risk (RR) of 16.4, 11.1, and 3.7, respectively, for sepsis.

**Table 4:** Multivariate logistic regression for factors associated with sepsis Odds ratios were estimated using logistic regression analysis AKI, acute kidney injury; CRP, C-reactive protein; HT, hyperthermia; χ^2^, chi-square

Parameter	Odds ratio	95% confidence interval	Test statistics	P-value
Male gender	1.8	0.5-5.8	χ^2^ = 1.73	0.95
Low birth weight	1.02	0.2-2.5	χ^2 ^= 0.003	0.3
Top fed	0.8	0.1-1.7	χ^2 ^= 0.01	0.9
Lethargy	16.4	2.5-108.1	χ^2 ^= 4.25	0.03
Repeat spike of HT	11.1	3.4-36.3	χ^2^ = 17.05	<0.001
Elevated CRP	3.7	1.2-11.3	χ^2 ^= 5.84	0.01
AKI	1.0	0.6-1.7	χ^2 ^= 0.00	1.0
Severe hyperthermia	0.88	0.5-1.42	χ^2 ^= 0.26	0.8

## Discussion

We studied 90 neonates who presented to us with fever and documented HT. Out of all the babies, 42.2% had sepsis, 34.5% had EH, and 23.3% had DF. Poor activity and a repeat episode of HT were clinical features significantly associated with sepsis. Elevated CRP was the only laboratory investigation that was significantly associated with the diagnosis of sepsis.

EH is common in a country such as India where the temperature and humidity are high [12,13]. Urban areas get very hot due to dense buildings, shrinking water bodies, and fewer trees. Admissions in the NICU due to heat-related illness increase during the summer months [5]. Common environmental reasons could be wrapping too many layers of clothes in hot and humid environments and leaving the baby in direct sunlight or in a parked car in hot weather. Babies admitted to the NICU may develop HT due to malfunctioning warmers, displaced probes, or phototherapy machines [14]. The incidence of EH in different studies may vary because of the climate of the place where they were conducted.

Babies in extreme summer months should be kept in a cool environment with light clothing, and frequent breastfeeding is encouraged. The WHO recommends that if the body temperature in a neonate is >40°C/104°F, the baby can be given a bath in tepid water (2°C lower than the baby’s temperature). The use of cold water or a cooler is dangerous [3].

Many Indian studies have shown a high incidence of DF, especially in the summer months [15,16]. DF is seen in term babies on exclusive breastfeeding and who may have hypernatremic dehydration [16,17]. The illness may be mild to severe with complications such as acute kidney injury (AKI), shock, cerebral hemorrhage, and thrombosis [10]. It is more common in the first week of life and in babies born to primipara mothers, especially by cesarean section [18]. Saxena et al. found that all the babies with DF were exclusively breastfed, 81.3% were born to primigravida mothers, and >50% had AKI [10]. We found DF in 23% of babies with HT; 24 out of 26 cases were seen in the summer months. In our study, the serum sodium levels and urea levels were significantly higher in babies with DF, and most cases (85%) had birth weights that were appropriate for gestation. Proper counseling in the antenatal period and after the birth of the baby about the proper technique of breastfeeding can reduce its incidence, and antibiotics have no role.

Bacterial sepsis may present with HT in neonates, but it is not a common symptom. The incidence of sepsis in febrile infants varies from 19% to 54% in various studies [19,20]. In a study by Naseh et al. on neonates admitted with HT, bacterial infection accounted for 37% of cases, EH accounted for 39% of cases, and 11% were due to viral illnesses such as upper respiratory tract infection and acute gastroenteritis [6]. Zarkesh et al. reviewed records of 202 febrile neonates, in which they found that only 19% of babies had serious bacterial infection (SBI) [21]. Brown et al. studied 69 febrile neonates, of whom 48 newborns did not have sepsis [22]. In babies aged <29 days, causes of fever other than sepsis were more prevalent in a study by Chang et al. [20]. The incidence of sepsis in our study was 42%.

Differentiating a febrile neonate with sepsis from other causes of HT is challenging. The clinicians rely on laboratory investigations and clinical findings to make a diagnosis. However, most laboratory investigations do not help in making a diagnosis. Routine blood parameters are seldom useful in differentiating bacterial sepsis from other benign causes. Chen et al., in an analysis of febrile admitted neonates, reported that CRP was significantly higher in the SBI group; total leukocyte count (TLC), neutrophil count, or band cells were not helpful in diagnosis [23]. In another study, febrile neonates with infection had higher CRP and thrombocytopenia; there was no significant difference in TLC, absolute neutrophil count (ANC), and the incidence of AKI between babies with and without infection [6].

Brown et al. studied 69 febrile neonates; they found that TLC had a moderate discriminatory power in identifying bacterial sepsis in newborns and concluded that the TLC threshold should not be used to identify bacterial infection in neonates [22]. The area under the curve (AUC) for the TLC prediction for sepsis was 0.72. (95% confidence interval {CI}: 0.5-0.87). In our study, CRP was the only investigation that was significantly different in the sepsis group and was associated with the diagnosis of sepsis.

Neonatal meningitis in term babies may have HT as a predominant symptom. Liu et al. reported that 97% of the patients with late-onset neonatal meningitis (onset of ≥7 days) and 84% of the patients with early-onset neonatal meningitis (onset of <7 days) had fever as a symptom [24]. We had 19 cases of meningitis, which accounted for 21% of all the babies. The incidence of culture-positive sepsis and clinical sepsis was 8% and 19%, respectively.

Different criteria have been used in the past to identify SBI in young infants, such as the Rochester criteria and Philadelphia protocol [22,25,26]. “Step by step” was another algorithm that was developed to identify a low-risk group of infants who can be safely managed as outpatients without antibiotics and lumbar puncture [27]. Most of the scores keep neonates in the high-risk category, except the Rochester criteria, which has no age defined for the low-risk group. These criteria apply to infants of varying ages from zero to 90 days and have been tested in a limited population. Low-risk criteria (LRC) as per the Rochester criteria had a negative predictive value of 98.4% to exclude SBI in a febrile neonate in a study by Zarkesh et al. [21].

A significant finding in our study was the association of the repeat episode of HT after admission with the diagnosis of sepsis. A repeat HT spike after admission had an RR of 11.1 (95% CI: 3.4-36.3) and a p-value of <0.001 for the diagnosis of sepsis. EH and DF are associated with high ambient temperatures and poor feeding practices. The temperature in the NICU is optimum, and the mother starts to feed the baby well when she is counselled about the proper technique of feeding. As a result, the baby does not develop a second episode of HT after admission in cases of EH and DF.

A universal approach toward the management of febrile neonates is lacking, and there is wide variation in protocols followed by different units [28]. There have been many efforts to develop an algorithm to aid in the approach to such babies. Naseh et al. proposed that all neonates with fever should be admitted, and those who are ill or do not have low-risk criteria should be administered systemic antibiotics immediately, and the neonates having low-risk criteria should be kept under close observation [6]. Many recommend hospitalizing all infants aged <90 days. The hospitalization of all infants has its own hazards and problems. The clinical prediction tools used in the past have shortcomings, as there is a lot of variability in the definition of SBI, and the cutoff for the various laboratory parameters was arbitrary. There are no universal guidelines regarding the management of febrile neonates.

The limitation of our study is that it is a single-center study. Also, we enrolled only admitted neonates. Many neonates with HT, especially those who were well-appearing, were not admitted by the parents and were followed in the outpatient department. The proportion of EH or DF may have been higher if these neonates were also included. The strengths of this study are that it is a prospective study and has an easy methodology, which can be reproduced easily in another setting.

## Conclusions

Although the risk of invasive bacterial infection is high in neonates, more than half of the neonates with HT may still have causes other than sepsis. Prompt antibiotic administration and complete workup are a must in sick babies with HT. For a well-appearing term neonate, a thorough workup for sepsis should be done, and systemic antibiotics should be administered only if the baby develops a repeat episode of HT after admission.

Routine workup and CSF analysis may not be warranted in all well-appearing term babies presenting with HT, especially in the presence of high environmental temperatures. All such neonates, nevertheless, must be kept under close observation. A practical scoring system for neonates should be developed to aid the clinician in making correct decisions. The healthcare workers and the family should be sensitized about the problem of EH and DF and ways to prevent and manage HT.
